# Cytoplasmic Skp2 Expression Is Increased in Human Melanoma and Correlated with Patient Survival

**DOI:** 10.1371/journal.pone.0017578

**Published:** 2011-02-28

**Authors:** Guangdi Chen, Yabin Cheng, Zhizhong Zhang, Magdalena Martinka, Gang Li

**Affiliations:** 1 Department of Dermatology and Skin Science, Jack Bell Research Centre, Vancouver Coastal Health Research Institute, University of British Columbia, Vancouver, British Columbia, Canada; 2 Department of Pathology, Jack Bell Research Centre, Vancouver Coastal Health Research Institute, University of British Columbia, Vancouver, British Columbia, Canada; University Medical Center Hamburg-Eppendorf, Germany

## Abstract

**Background:**

S-phase kinase protein 2 (Skp2), an F-box protein, targets cell cycle regulators via ubiquitin-mediated degradation. Skp2 is frequently overexpressed in a variety of cancers and associated with patient survival. In melanoma, however, the prognostic significance of subcellular Skp2 expression remains controversial.

**Methods:**

To investigate the role of Skp2 in melanoma development, we constructed tissue microarrays and examined Skp2 expression in melanocytic lesions at different stages, including 30 normal nevi, 61 dysplastic nevi, 290 primary melanomas and 146 metastatic melanomas. The TMA was assessed for cytoplasmic and nuclear Skp2 expression by immunohistochemistry. The Kaplan-Meier method was used to evaluate the patient survival. The univariate and multivariate Cox regression models were performed to estimate the harzard ratios (HR) at five-year follow-up.

**Results:**

Cytoplasmic but not nuclear Skp2 expression was gradually increased from normal nevi, dysplastic nevi, primary melanomas to metastatic melanomas. Cytoplasmic Skp2 expression correlated with AJCC stages (I vs II–IV, *P*<0.001), tumor thickness (≤2.00 vs >2.00 mm, *P*<0.001) and ulceration (*P* = 0.005). Increased cytoplasmic Skp2 expression was associated with a poor five-year disease-specific survival of patients with primary melanoma (*P* = 0.018) but not metastatic melanoma (*P*>0.05).

**Conclusion:**

This study demonstrates that cytoplasmic Skp2 plays an important role in melanoma pathogenesis and its expression correlates with patient survival. Our data indicate that cytoplasmic Skp2 may serve as a potential biomarker for melanoma progression and a therapeutic target for this disease.

## Introduction

Cutaneous melanoma is an aggressive cancer type originating from melanocytes in the human skin [1], [2]. Although early diagnosed melanoma is curable with surgical excision, up to 20% of patients will develop metastatic tumors due to its high capability of invasion and rapid metastasis to other organs [3], [4]. Despite many advances in cancer treatment over the last several decades, the prognosis for patients with advanced melanoma remains poor. The 5-year survival rate for patients with distant metastases is less than 10% [5].

The main hallmark of cancer is uncontrolled cellular proliferation with alterations in the expression or activity of proteins that are intimately involved in cell cycle regulation, differentiation and apoptosis [6]. These processes are regulated by transcription, translation, post-translational modifications and degradation of key regulatory proteins, and as such, the ubiquitin–proteasome system has a crucial role in maintaining and regulating cellular homeostasis [7]. In the ubiquitin–proteasome degradation pathway, the ubiquitin is transferred and covalently attached to substrates by the sequential action of three enzymes, namely E1 (ubiquitin-activating enzyme), one of many E2s (ubiquitin-conjugating enzymes) and one of many E3s (ubiquitin ligases) [8]. The E3 ligases are mainly classified into two classes on the basis of structural similarities: the RING-finger proteins and the HECT-domain proteins [7]. Most of RING-finger type ubiquitin ligases contain a cullin (Cul) protein subunit, which were also named as cullin RING ubiquitin ligases (CRLs) [9]. The SCF (Skp1-Cul1-F-box protein) ubiquitin ligases are the best characterized mammalian CRLs, and the F-box protein provides the substrate targeting specificity of the complex [10]. Till now, sixty-nine human F-box proteins that have been identified, and among them, S-phase kinase-associated protein 2 (Skp2) has emerged as a key regulator in different cellular processes [7], [11]. Skp2 is an oncogenic protein that targets tumor suppressor proteins for degradation, including cyclin-dependent kinase (CDK) inhibitors p21^Cip1^, p27^Kip1^ and p57^Kip2^ [7], [11]. Increased levels of Skp2 and reduced levels of p27 are observed in many types of cancer, and these levels are used as independent prognostic markers in several cases [12].

In melanoma, Li *et al*. have shown that gain of Skp2 protein expression are implicated in melanoma progression and Skp2 cytoplasmic expression predicted worse 10-year overall survival in primary melanoma, suggesting that cytoplasmic expression of Skp2 defines a subset of aggressive melanomas [13]. The study by Woenckhaus *et al*. suggests that Skp2 could contribute to melanoma progression and vertical growth phase (VGP) melanomas show significant higher nuclear Skp2 expression when compared with the radial growth phase (RGP) [14]. However, they found that the nuclear but not the cytoplasmic Skp2 expression correlated with a reduced survival time in melanoma [14]. These studies indicate that Skp2 expression plays a key role in melanoma development, but the correlation between the sub-cellular Skp2 expression and melanoma patient survival remains contradictory which may be due to small sample size in their studies. To further examine the role of Skp2 subcellular expression in melanoma development and its prognostic significance, we evaluated both nuclear and cytoplasmic staining in 30 normal nevi, 61 dysplastic nevi, 290 primary melanomas and 146 metastatic melanomas.

## Materials and Methods

### Ethics statement

The use of human skin tissues and the waiver of patient consent in this study were approved by the Clinical Research Ethics Board of the University of British Columbia. The study was conducted according to the principles expressed in the Declaration of Helsinki.

### Patient specimens and tissue microarray construction

TMA construction and immunohistochemistry of TMA were described elsewhere [15], [16]. Briefly, formalin-fixed, paraffin-embedded tissues from 49 normal nevi, 100 dysplastic nevi, 403 primary melanomas, and 161 metastatic melanomas were used for our present study. All specimens were obtained from the 1990 to 2009 archives of the Department of Pathology, Vancouver General Hospital. The most representative tumor area was carefully selected and marked on the hematoxylin and eosin-stained slide. The TMAs were assembled using a tissue-array instrument (Beecher Instruments, Silver Spring, MD). Duplicate 0.6-mm-thick tissue cores were taken from each biopsy specimen. Multiple 4-µm sections were cut with a Leica microtome (Leica Microsystems Inc, Bannockburn, IL) and transferred to adhesive-coated slides. One section from each TMA was routinely stained with hematoxylin and eosin. The remaining sections were stored at room temperature for immunohistochemical staining.

### Immunohistochemistry of TMA

TMA slides were dewaxed at 55°C for 30 min followed by three 5-min washes with xylene. The rehydration of tissues was performed by 5-min washes in 100, 95, and 80% ethanol and distilled water. Antigen retrieval was performed by heating the samples at 95°C for 30 min in 10 mM sodium citrate (pH 6.0). Endogenous peroxidase activity was blocked by incubation in 3% hydrogen peroxide for 30 min. After 30 min blocking with the universal blocking serum (Dako Diagnostics, Carpinteria, CA), the sections were incubated with monoclonal mouse anti-Skp2 antibody (1∶100 dilution; clone A-2; Santa Cruz Biotechnology, Santa Cruz, CA) at 4°C overnight. The sections were then incubated for 30 min each with a biotin-labeled secondary antibody and then streptavidin-peroxidase (Dako Diagnostics). The samples were developed using 3,3′-diaminobenzidine substrate (Vector Laboratories, Burlington, Ontario, Canada) and counterstained with hematoxylin. Dehydration was then performed following a standard procedure and the slides were sealed with coverslips. Negative controls were performed by omitting the Skp2 antibody during the primary antibody incubation.

### Evaluation of immunostaining

Positive Skp2 immunostaining is defined as either cytoplasmic or nuclear staining and graded according to both intensity and percentage of cells with positive staining. The evaluation of Skp2 staining was blindly and independently examined by two observers, including one dermatopathologist. In few cases with discrepancy, between the two observers, the immunostained slides were reviewed in a double viewing microscope so that the discrepancy was settled. Skp2 staining intensity was scored as 0, 1+, 2+, and 3+. The percentage of Skp2-positive cells was also scored into 4 categories: 1 (0–25%), 2 (26–50%), 3 (51–75%), and 4 (76–100%). In the cases with a discrepancy between duplicated cores, the average score from the two tissue cores was taken as the final score. The level of Skp2 staining was evaluated by immunoreactive score (IRS) [17], which is calculated by multiplying the scores of staining intensity and the percentage of positive cells. Based on the IRS, Skp2 staining pattern was defined as low staining (0–6) and high staining (8–12).

### Statistical analysis

Differences in demographic and clinical characteristics and expression levels of Skp2 were evaluated by χ^2^ tests between patient subgroups. Survival time was calculated from the date of melanoma diagnosis to the date of death or last follow-up. The Kaplan-Meier method and log-rank test were used to evaluate the effects of Skp2 expression on the overall and disease-specific survival of patients. Univariate or multivariate Cox proportional hazards regression models were preformed to estimate the crude hazard ratios (HRs) or adjusted HRs and their 95% confidential intervals (CIs). SPSS version 11.5 (SPSS Inc, Chicago, IL) software was used for all analyses.

## Results

### Increased cytoplasmic Skp2 expression in melanoma

**Figure 1:**
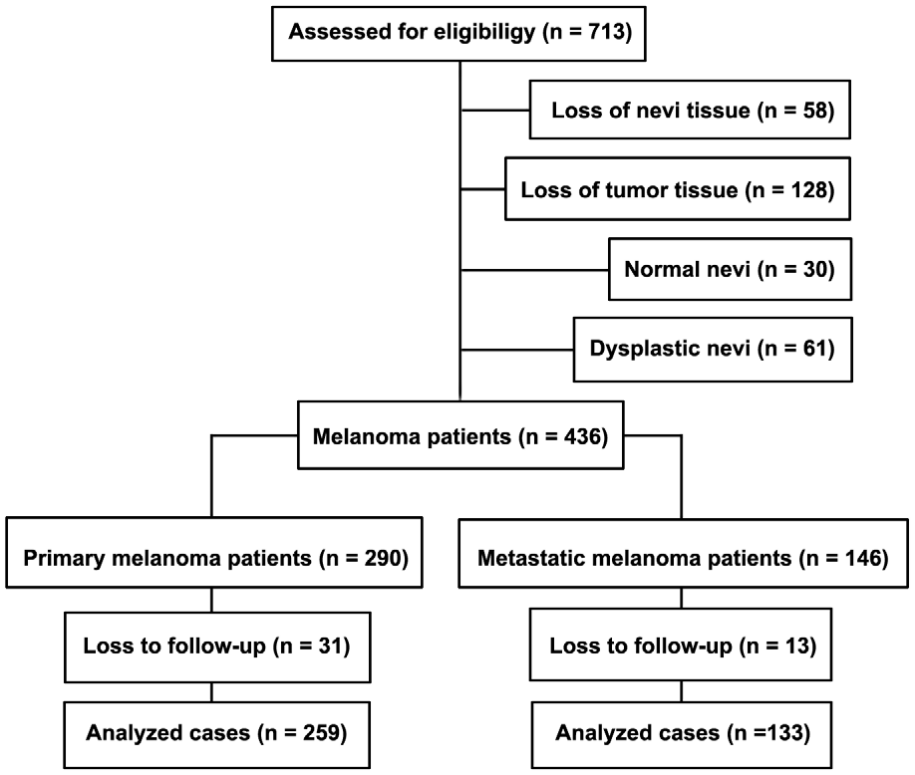
CONSORT diagram for patient inclusion and exclusion.

We investigated Skp2 expression in human melanocytic biopsies using TMA and immunohistochemistry. A total of 713 patients were enrolled. Due to loss of biopsy cores or insufficient tumor cells present in the cores, 30 normal nevi, 61 dysplastic nevi, 290 primary melanomas and 146 metastatic melanomas could be evaluated for Skp2 staining and included in the present study (Figure 1). For the 436 melanoma cases, there were 258 men and 178 women, with age ranging from 7 to 95 years (median, 60 years). 167 tumors were at AJCC stage I, 123 at stage II, 59 at stage III, and 87 at stage IV. Among the 290 primary melanoma cases, 176 tumors were ≤2.00 mm, and 114 tumors were >2.00 mm. Ulceration was observed in 53 cases. Seventy-one melanomas were located in sun-exposed sites (head and neck), and 219 were located in sun-protected sites (other locations) (Table 1).

**Table 1:** Cytoplasmic Skp2 staining and clinicopathological characteristics of melanomas.

Variables	Cytoplasmic Skp2 Staining			
	Low staining	High staining	Total	*P* †
*All melanoma (n = 436)*				
Age (years)				
≤60	126 (56.3%)	98 (43.7%)	224 (51.4%)	0.133
>60	104 (49.1%)	108 (50.9%)	212 (48.6%)	
Sex				
Male	135 (52.3%)	123 (47.7%)	258 (52.8%)	0.830
Female	95 (53.4%)	83 (46.6%)	178 (47.2%)	
AJCC stage				
I	112 (67.1%)	55 (32.9%)	167 (38.3%)	<0.001‡
II	54 (43.9%)	69 (56.1%)	123 (28.2%)	0.958§
III	25 (42.4%)	34 (57.6%)	59 (13.5%)	
IV	39 (44.8%)	48 (55.2%)	87 (20.0%)	
*Primary melanoma (n = 290)*				
Age (years)				
≤60	84 (60.4%)	55 (39.6%)	139 (47.9%)	0.292
>60	82 (54.3%)	69 (45.7%)	151 (52.1%)	
Sex				
Male	89 (56.3%)	69 (43.7%)	158 (54.5%)	0.731
Female	77 (58.3%)	55 (41.7%)	132 (45.5%)	
Tumor thickness (mm)				
≤2.00	118 (67.0%)	58 (33.0%)	176 (60.7%)	<0.001
>2.00	48 (42.1%)	66 (57.9%)	114 (39.3%)	
Ulceration				
Absent	141 (59.5%)	96 (40.5%)	237 (81.7%)	0.005
Present	25 (47.2%)	28 (52.8%)	53 (18.3%)	
Site*				
Sun-protected	124 (56.6%)	95 (43.4%)	219 (75.5%)	0.707
Sun-exposed	42 (59.2%)	29 (40.8%)	71 (24.5%)	
*Metastatic melanoma (n = 146)*				
Age (years)				
≤60	42 (49.4%)	43 (50.6%)	85 (58.2%)	0.109
>60	22 (36.1%)	39 (63.9%)	61 (41.8%)	
Sex				
Male	46 (46.0%)	54 (54.0%)	100 (68.5%)	0.437
Female	18 (39.1%)	28 (60.9%)	46 (31.5%)	

*Sun-protected sites: trunk, arm, leg and feet; Sun-exposed sites: head and neck.

† χ^2^ test.

‡ Comparison between all AJCC stages.

§ Comparison between AJCC stage II, III and IV.

**Figure 2:**
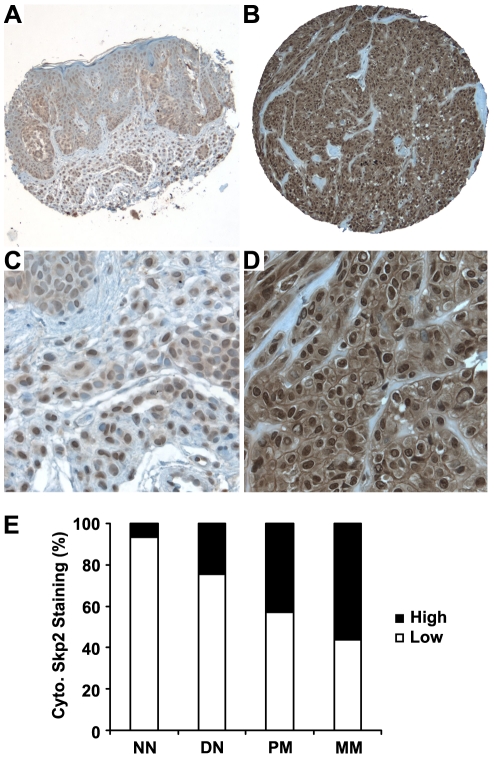
Cytoplasmic Skp2 expression is increased in human melanomas. Representative images of cytoplasmic Skp2 immunohistochemical staining in human melanocytic lesions. (A and C) Low cytoplasmic Skp2 staining, (B and D) High cytoplasmic Skp2 staining. (E) Cytoplasmic Skp2 expression is increased from normal nevi to dysplastic nevi and melanoma. Significant differences for Skp2 staining are observed between normal and dysplastic nevi (*P* = 0.039, χ^2^ test), dysplastic nevi and primary melanoma (*P* = 0.008, χ^2^ test), and primary and metastatic melanoma (*P* = 0.008, χ^2^ test). NN, normal nevi; DN: dysplastic nevi; PM: primary melanoma; MM: metastatic melanoma. Magnification: ×100 for A and B; ×400 for C and D.

As shown in Figure 2 and Supporting Information Figure S1, various levels of cytoplasmic and nuclear Skp2 staining were observed in nevi and melanoma biopsies. The Skp2 staining was found consistent when two different antibodies were used in a small TMA including 20 cases of nevi and 20 case of melanomas (Supporting Information Figure S2). There are differences in the pattern of cytoplasmic Skp2 expression in melanocytic lesions at different stage, with increased levels of expression from normal nevi to dysplastic nevi and melanoma. Significant differences for Skp2 staining were observed between normal and dysplastic nevi (*P* = 0.039, χ^2^ test), dysplastic nevi and primary melanoma (*P* = 0.008, χ^2^ test), and primary and metastatic melanoma (*P* = 0.008, χ^2^ test) (Figure 2E). However, there was no significant difference for nuclear Skp2 staining among different stages of melanocytic lesions (*P*>0.05, χ^2^ test) (Figure S1).

### Cytoplasmic Skp2 expression correlates with AJCC stages, tumor thickness and ulceration

**Figure 3:**
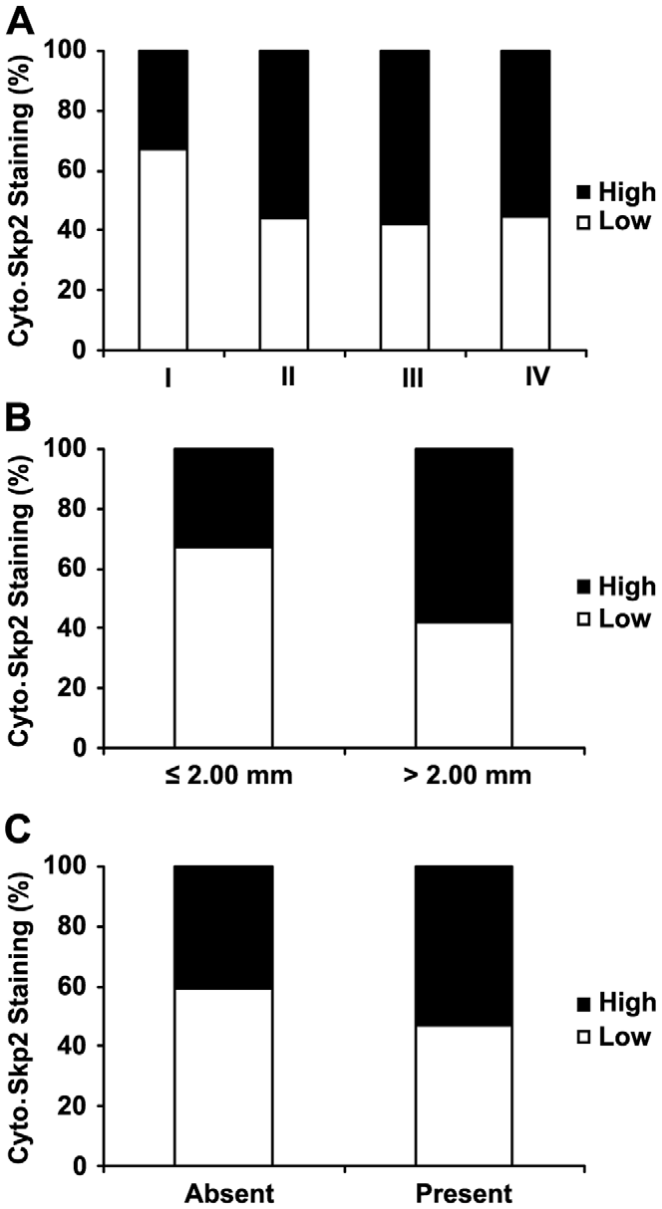
Cytoplasmic Skp2 correlates with melanoma AJCC stages, tumor thickness and ulceration. (A) Tumors in AJCC stages II, III and IV have a higher percentage of high cytoplasmic Skp2 expression compared with tumors in stage I (*P*<0.001, χ^2^ test). (B) Tumors thicker than 2.0 mm have a higher percentage of high cytoplasmic Skp2 expression compared with tumors ≤2.0 mm thick (*P*<0.001, χ^2^ test). (C) Increased cytoplasmic Skp2 expression is correlated with ulceration of melanoma (*P* = 0.005, χ^2^ test).

Since cytoplasmic Skp2 expression was correlated with melanoma progression, we next examined the correlation between cytoplasmic Skp2 expression and different clinicopathologic characters. As shown in Figure 3A and Table 1, high expression of cytoplasmic Skp2 was detected in 33% of melanomas at AJCC stage I compared to only 55–58% of melanomas at AJCC II, III and IV (*P*<0.001, χ^2^ test); however, no significant difference was found in Skp2 expression between AJCC stage II and III or IV, indicating that increased cytoplasmic Skp2 expression may be involved in the melanoma development from stage I to II. In addition, we found that the cytoplasmic Skp2 expression was correlated with Ki67 expression, the proliferative index (Supporting Information Figure S3), and inversely correlated with nuclear p27 expression, a cyclin-dependent kinase inhibitor for cell cycle progression (Supporting Information Figure S4). These data suggest that cytoplasmic Skp2 regulates melanoma progression may through promoting melanoma cell cycle progression.

There was no correlation between cytoplasmic Skp2 expression and age or sex in all melanoma patients (Table 1). In primary melanoma, high cytoplasmic Skp2 expression was found in 58% of tumors with thickness >2.00 mm, compared to 33% of melanomas with thickness ≤2.00 mm (*P*<0.001, χ^2^ test; Figure 3B). Increased cytoplasmic Skp2 expression was correlated with ulceration of melanoma (*P* = 0.005, χ^2^ test; Table 1). We did not find significant correlations between cytoplasmic Skp2 expression and other variables (Table 1). In addition, cytoplasmic Skp2 expression was not correlated with age or sex in metastatic melanoma (Table 1).

### Increased cytoplasmic Skp2 expression is associated with poor patient survival

**Figure 4:**
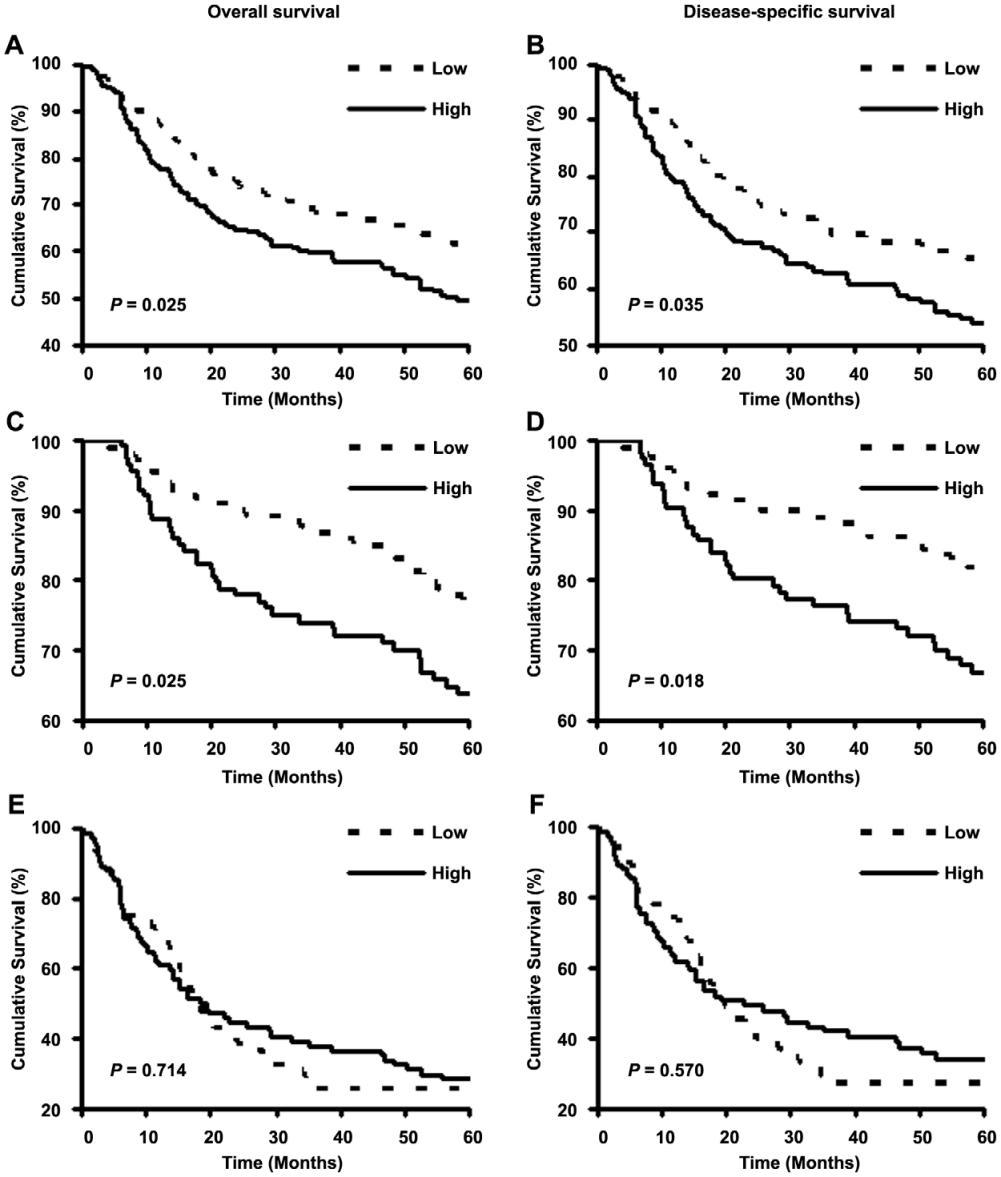
Cytoplasmic Skp2 expression is associated with five-year survival of all melanoma and primary melanoma patients. Kaplan-Meier curves analyses for the correlation between cytoplasmic Skp2 expression and overall or disease-specific five-year survival in all melanoma patients (A,B), primary melanoma patients (C,D) and metastatic melanoma patients (E,F).

A total of 392 patients had complete follow-up and clinical information (Figure 1). Demographic and clinical characteristics of the patients were listed in Table 2 and Supporting Information Table S1 and S2. The Kaplan-Meier curve and log-rank test analyses revealed that increased cytoplasmic Skp2 expression was associated with poor overall (*P* = 0.025) or disease-specific five-year survival (*P* = 0.035) (Figure 4A and B). Next, we separated all melanoma (n = 392) into primary melanoma (n = 259) and metastatic melanoma (n = 133) and found cytoplasmic Skp2 expression was associated with overall (*P* = 0.025) and disease-specific five-year survival (*P* = 0.018) in primary melanoma patients (Figure 4C and D). However, cytoplasmic Skp2 expression was not associated with overall and disease-specific five-year survival in metastatic melanoma patients (*P*>0.05 for both) (Figure 4E and F). Since previous study showed that nuclear Skp2 expression was associated with melanoma patient survival [14], we also examined the correlation between nuclear Skp2 expression and patient survival. The results indicate that nuclear Skp2 expression is not associated with overall and disease-specific five-year survival in all melanoma, primary melanoma or metastatic melanoma patients (Supporting Information Figure S5).

**Table 2:** Univariate Cox proportional regression analysis on overall and disease-specific 5-year survival of 259 primary melanoma patients.

Variable	Patients (%)	Overall survival				Disease-specific survival			
		Deaths	Death Rate	HR (95% CI)	*P* †	Deaths	Death Rate	HR (95% CI)	*P* †
Age									
≤60	124 (47.9%)	21	16.9%	2.65 (1.58–4.42)	<0.001	21	16.9%	2.14 (1.26–3.65)	0.005
>60	135 (52.1%)	48	35.6%			39	28.9%		
Sex									
Male	138 (53.3%)	37	26.8%	1.00 (0.62–1.61)	0.994	34	24.6%	0.89 (0.53–1.48)	0.645
Female	121 (46.7%)	32	26.4%			26	21.5%		
Thickness (mm)									
≤2.00	152 (58.7%)	22	14.5%	4.32 (2.60–7.19)	<0.001	17	11.2%	5.09 (2.89–8.94)	<0.001
>2.00	107 (41.3%)	47	43.9%			43	40.2%		
Ulceration									
Absent	211 (85.3%)	42	19.9%	4.64 (2.84–7.59)	<0.001	37	17.5%	4.43 (2.61–7.51)	<0.001
Present	48 (14.7%)	27	56.3%			23	47.9%		
Site*									
Sun-protected	196 (75.7%)	53	27.0%	1.00 (0.57–1.76)	0.990	44	22.4%	1.21 (0.68–2.14)	0.515
Sun-exposed	63 (24.3%)	16	25.4%			16	25.4%		
Cytoplasmic Skp2									
Low staining	145 (56.0%)	30	20.7%	1.71 (1.07–2.76)	0.027	25	17.2%	1.84 (1.10–3.08)	0.020
High staining	114 (44.0%)	39	34.2%			35	30.7%		
Nuclear Skp2									
Low staining	60 (23.2%)	21	35.0%	0.65 (0.39–1.09)	0.100	19	31.7%	0.62 (0.36–1.06)	0.080
High staining	199 (76.8%)	48	24.1%			41	20.6%		

*Sun-protected sites: trunk, arm, leg and feet; Sun-exposed sites: head and neck.

† Log-Rank test. Abbreviations: HR, hazard ratio; CI, confidence interval.

Next, we used univariate Cox proportional hazards regression model to estimate the crude hazard ratios (HRs) of Skp2 expression or each clinicopathologic variable on patient survival. The log-rank test and univariate Cox regression analyses revealed that both AJCC stages and cytoplasmic Skp2 expression were significantly associated with overall or disease-specific survival in all melanoma patients (Supporting Information Table S1). In primary melanoma patients, age, tumor thickness, ulceration and cytoplasmic Skp2 expression were all significantly associated with both overall and disease-specific survival (Table 2). However, the nuclear expression was not associated with either overall or disease-specific survival outcomes (Table 2). In metastatic melanoma patients, both cytoplasmic and nuclear expressions were not associated with either overall or disease-specific survival (Supporting Information Table S2).

### Cytoplasmic Skp2 expression is not an independent prognostic marker for primary melanoma

Since cytoplasmic Skp2 was associated with melanoma patient survival, we next examined whether cytoplasmic Skp2 expression is an independent prognostic marker for melanoma patient survival by multivariate Cox proportional hazard analysis. Age, sex, tumor thickness, ulceration and location and cytoplasmic Skp2 expression were included in the regression model. The results indicated that cytoplasmic Skp2 expression was not associated with both overall and disease-specific survival (*P*>0.05 for both, Table 3). As expected, cytoplasmic Skp2 expression was not associated with survival in all melanoma patients by adjustment with AJCC stages, age and sex (Supporting Information Table S3). These data indicate that cytoplasmic Skp2 staining is not independent prognostic factor for five-year survival of melanoma patients.

**Table 3:** Multivariate Cox regression analysis on overall and disease-specific 5-year survival of 259 primary melanoma patients.

Variables*	Overall survival					Disease-specific survival				
	β†	SE	HR	95% CI	*P* ‡	β†	SE	HR	95% CI	*P* ‡
Age	0.544	0.278	1.74	1.01–3.02	0.046	0.282	0.289	1.33	0.75–2.34	0.329
Sex	0.012	0.254	1.01	0.62–1.66	0.964	–0.060	0.276	0.94	0.55–1.62	0.828
Thickness	1.035	0.295	2.82	1.58–5.02	<0.001	1.241	0.324	3.46	1.83–6.53	<0.001
Ulceration	0.908	0.282	2.48	1.43–4.31	0.001	0.884	0.301	2.42	1.34–4.37	0.003
Location	–0.091	0.292	0.91	0.52–1.62	0.755	0.100	0.301	1.11	0.61–2.00	0.740
Cytoplasmic Skp2	0.124	0.258	1.13	0.68–1.88	0.629	0.177	0.277	1.19	0.69–2.05	0.523

*Coding of variables: Age was coded as 1 (≤60 years) and 2 (>60 years). Sex was coded as 1 (male) and 2 (female). Thickness was coded as 1 (≤2.00 mm) and 2 (>2.00 mm). Ulceration was coded as 1 (absent) and 2 (present). Location was coded as 1 (sun-protected) and 2 (sun-exposed). Cytoplasmic Skp2 was coded as 1 (low staining) and 2 (high staining).

† β: regression coefficient.

‡ Log-Rank test.

Abbreviations: SE, standard error of β; HR, hazard ratio; CI, confidence interval.

## Discussion

Melanocytes are located in the skin, eyes and other epithelial tissues and are the precursors of benign nevus and melanoma [18], [19]. The highly differentiated benign nevi have a low proliferation potential and retain melanin production, whereas malignant melanoma show increased proliferation and may have partly or fully lost the ability to synthesize melanin, in parallel with loss of differentiation [19]. Constitutive activation of cell proliferation signaling, including mitogen-activated protein kinase (MAPK) pathway, induces malignant transformation of melanocytes to melanoma [20]. Due to their relatively small size, variable criteria for histological diagnosis, controversial terminology, and difficulty in establishing *in vitro* cultures, melanocytic dysplastic nevi have hardly been studied [21]. The limited studies on dysplastic nevi mainly focus on some genetic changes [22]. In addition to genetic changes, recent studies indicate that the alterations of tumor suppressor, onco-proteins and growth factors contribute to development of dysplastic nevi from normal nevi and suggest that evolution of some dysplastic nevi may result in primary melanoma [23], [24]. Our study showed that cytoplasmic Skp2 expression was gradually increased from normal nevi, dysplastic nevi to melanoma tissues, indicating that increased cytoplasmic Skp2 expression may be involved in the development of dysplastic nevi and melanoma. Skp2 has oncogenic potential and is overexpressed in human cancers [25]. To our knowledge, this study for the first time showed that cytoplasmic Skp2 plays a key role in both melanoma initiation and progression.

Mutations in BRAF, a regulator of the mitogen-activated protein-kinase kinase (MAPKK)-ERK1/2 pathway, are associated with approximately 70% of melanomas; the most common mutants are BRAF^V599E^ and BRAF^V600E^ [26], [27]. In melanoma cells, mutation in BRAF constitutively activates BRAF signaling, which regulates Cks1/Skp2-mediated p27 degradation and controls G1 cell cycle event [28], [29]. In human thyroid carcinomas, constitutive signaling of the MAP kinase cascade contributes to the development of thyroid cancer promoted by activated RAS and BRAF onco-proteins and that this occurs, at least in part, by compromising the Skp2-dependent degradation pathway [30]. Our study showed that cytoplasmic Skp2 expression was increased in melanoma, which may be due to BRAF mutation. In addition, increased copy number at the Skp2 locus might also be associated with overexpression of Skp2 protein in human metastatic melanoma tissues [31].

In primary melanoma, we found a correlation between the depth of tumor invasion (thickness), ulceration and cytoplasmic Skp2 expression in the primary melanoma, which suggests a role of Skp2 in tumor invasion. It has been reported that overexpression of Skp2 correlated with metastasis in different cancers including colorectal tumors, lung cancers, oral and esophageal squamous cell carcinoma, human gastric carcinoma and pancreatic ductal adenocarcinoma [32], [33], . Skp2 regulates invasive ability of melanoma cells [40] and overexpression of Skp2 correlates with advanced melanoma [31]. Li *et al*. found that Skp2 phosphorylation by Akt triggers SCF complex formation and E3 ligase activity, promotes cell proliferation and tumorigenesis [41]. Akt-mediated phosphorylation triggers 14-3-3 beta-dependent Skp2 relocalization to the cytosol and positively regulates cell migration. Furthermore, high levels of activation of Akt correlate with the cytosolic accumulation of Skp2 in human colonic adenocarcinoma. In addition, Skp2 overexpression induces the expression of matrix metalloproteinase (i.e. MMP-2, MMP-9) and invasion of lung cancer cells [42]. Thus, increased cytoplasmic Skp2 expression contributes to melanoma invasion possibly through multiple pathways since both Akt and MMP proteins play import roles in melanoma invasion and metastases [16], [43], [44].

Cytoplasmic but not nuclear Skp2 expression was associated with five-year overall and disease-specific survival in primary melanoma patients. This finding was consistent with the study by Li *et al*. that high cytoplasmic but not nuclear Skp2 expression predicted worse 10-year overall survival in primary melanoma [13]. However, Woenckhaus *et al*. found that increased nuclear Skp2 expression correlates with a reduced survival time in melanoma [14]. The controversial findings may be due to a relative small number of patients (n = 25) for the survival analysis in the study by Woenckhaus *et al*. Recent study by Rose *et al*. showed that high Skp2 expression (>25% Skp2 staining) predicted worse seven-year post-recurrence survival when comparing low Skp2 expression (≤25% Skp2 staining) in 93 cases of metastatic melanoma patients [31]. In our study, neither nuclear nor cytoplasmic Skp2 expression was associated with five-year survival in 133 cases of metastatic melanoma patients. The clarification on the correlation between Skp2 expression and survival in metastatic melanoma patients awaits future study with a longer follow-up time.

Although surgical removal has been reported with cure rate over 80% for stage I melanoma and as high as 98% for melanoma-in-situ [45], [46], there is no effective treatment for advanced melanoma, It is important to understand the molecular mechanisms of melanoma development and provides a necessary basis to enable the generation of more effective therapeutic modalities. We found that cytoplasmic Skp2 expression is increased in melanoma and the increased cytoplasmic Skp2 expression correlated with melanoma development. In melanoma, Skp2 plays a key role in promoting cell cycle progression [47], [48], [49], [50]. Katagiri *et al*. reported that knockdown of Skp2 inhibited the melanoma cell growth *in vitro* and suppressed tumor proliferation *in vivo*, suggesting that gene silencing of Skp2 can be a potential tool of cancer gene therapy in malignant melanoma [51]. Another study by Sumimoto *et al*. revealed that the combined suppression of BRAF^V599E^ and Skp2 inhibited cell growth and attenuated the invasive potential of melanoma cell lines *in vitro* prompting speculation regarding the possibility of combination therapy targeting BRAF and Skp2 [40]. Thus, targeting Skp2 in gene therapy may hold promise for melanoma treatment.

## Supporting Information

Figure S1 Nuclear Skp2 expression does not correlate with human melanoma progression.
(DOCX)

Figure S2 Skp2 staining was consistent when probed by two different anti-Skp2 antibodies.
(DOCX)

Figure S3 Cytoplasmic Skp2 expression was positively correlated with Ki67 expression.
(DOCX)

Figure S4 Cytoplasmic Skp2 expression was inversely correlated with nuclear p27 expression.
(DOCX)

Figure S5 Nuclear Skp2 expression is not associated with melanoma patient survival.
(DOCX)

Table S1 Univariate Cox proportional regression analysis on overall and disease-specific 5-year survival of all 392 melanoma patients.
(DOC)

Table S2 Univariate Cox proportional regression analysis on overall and disease-specific 5-year survival of 133 patients with metastic melanoma.
(DOC)

Table S3 Multivariate Cox regression analysis on overall and disease-specific 5-year survival of all 392 melanoma patients.
(DOC)
