# Supplementary material for: Cytoplasmic Skp2 Expression Is Increased in Human Melanoma and Correlated with Patient Survival
Source: PLoS One. 2011 Feb 28;6(2):e17578. doi: 10.1371/journal.pone.0017578 (PMC3046256; doi:10.1371/journal.pone.0017578)

**Figure S1.** Nuclear Skp2 expression does not correlate with human melanoma progression. Representative images of nuclear Skp2 immunohistochemical staining in human melanocytic lesions. (A and C) Low nuclear Skp2 staining, (B and D) High nuclear Skp2 staining. (E) No significant difference for nuclear Skp2 staining is observed among normal nevi, dysplastic nevi, primary melanoma and metastatic melanoma (*P* > 0.05, χ^2^ test). NN, normal nevi; DN: dysplastic nevi; PM: primary melanoma; MM: metastatic melanoma. Magnification: ×100 for A and B; ×400 for C and D.


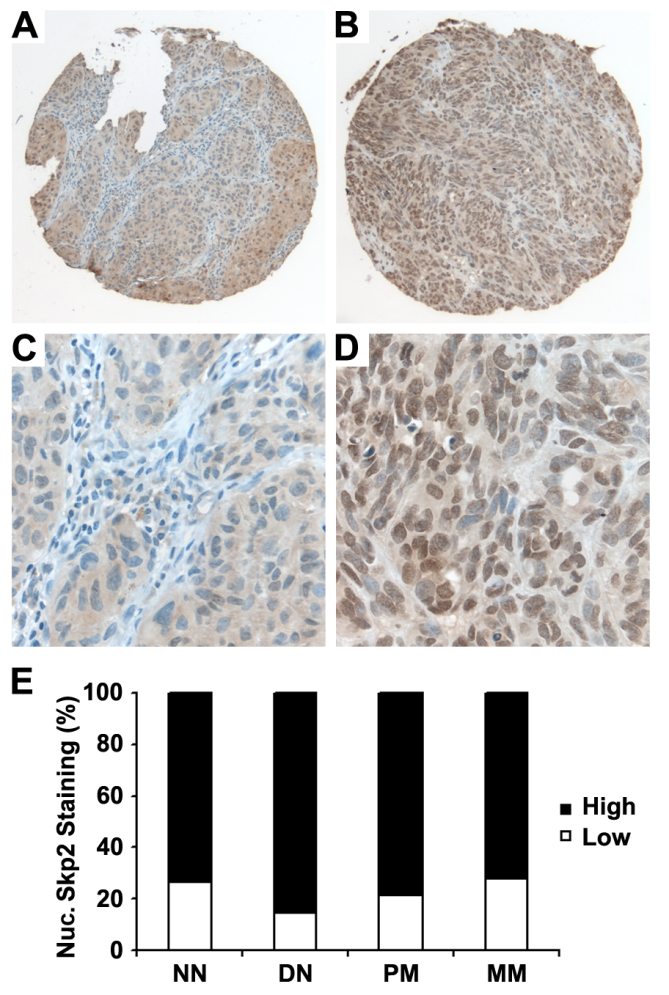

Supplement: Figure S1 — Nuclear Skp2 expression does not correlate with human melanoma progression. (DOCX) [file pone.0017578.s001.docx]
