# Supplementary material for: Cytoplasmic Skp2 Expression Is Increased in Human Melanoma and Correlated with Patient Survival
Source: PLoS One. 2011 Feb 28;6(2):e17578. doi: 10.1371/journal.pone.0017578 (PMC3046256; doi:10.1371/journal.pone.0017578)

**Figure S2.** Skp2 staining was consistent when probed by two different anti-Skp2 antibodies. Representative images of Skp2 immunohistochemical staining in human melanocytic lesions by anti-Skp2 (clone A-2, 1:100 dilution; Santa Cruz Biotechnology, Santa Cruz, CA) antibody (A and C), and by anti-Skp2 (clone N-19, 1:50 dilution; Santa Cruz) antibody (B and D). Low Skp2 staining in nevi (A and B); High Skp2 staining in melanoma (C and D). Pearson correlations were calculated between Skp2 staining (n=23) by these two antibodies and the coefficients were calculated for Skp2 overall staining (r=0.477, *P*=0.025), Skp2 nuclear staining (r=0.608, *P*=0.002) and Skp2 cytoplasmic staining (r=0.462, *P*=0.030). Magnification: ×100.


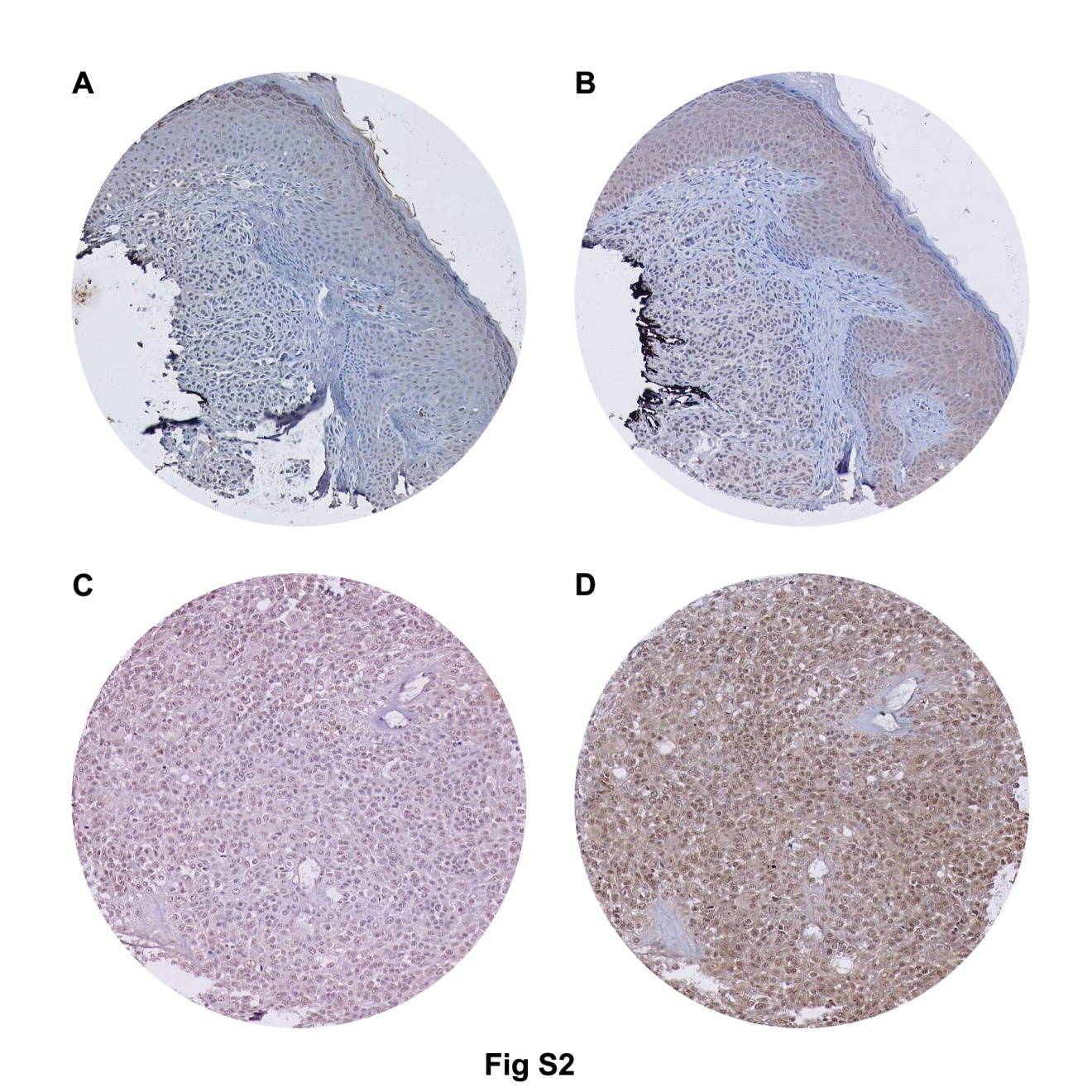

Supplement: Figure S2 — Skp2 staining was consistent when probed by two different anti-Skp2 antibodies. (DOCX) [file pone.0017578.s002.docx]
