# Supplementary material for: Cytoplasmic Skp2 Expression Is Increased in Human Melanoma and Correlated with Patient Survival
Source: PLoS One. 2011 Feb 28;6(2):e17578. doi: 10.1371/journal.pone.0017578 (PMC3046256; doi:10.1371/journal.pone.0017578)

**Figure S3.** Cytoplasmic Skp2 expression was positively correlated with Ki67 expression. Representative images of cytoplasmic Skp2 (clone A-2, 1:100 dilution; Santa Cruz) (A and C) and Ki67 (monoclonal antibody from Thermal Scientific, Fremont, CA) (B and D) immunohistochemical staining in human melanocytic lesions. Low cytoplasmic Skp2 staining and Ki67 in nevi (A and B); High cytoplasmic Skp2 and Ki67 staining in melanoma (C and D). Pearson correlation was calculated between cytoplasmic Skp2 and Ki67 staining (n=23) and the coefficient was calculated (r=0.436, *P*=0.037). Magnification: ×100.


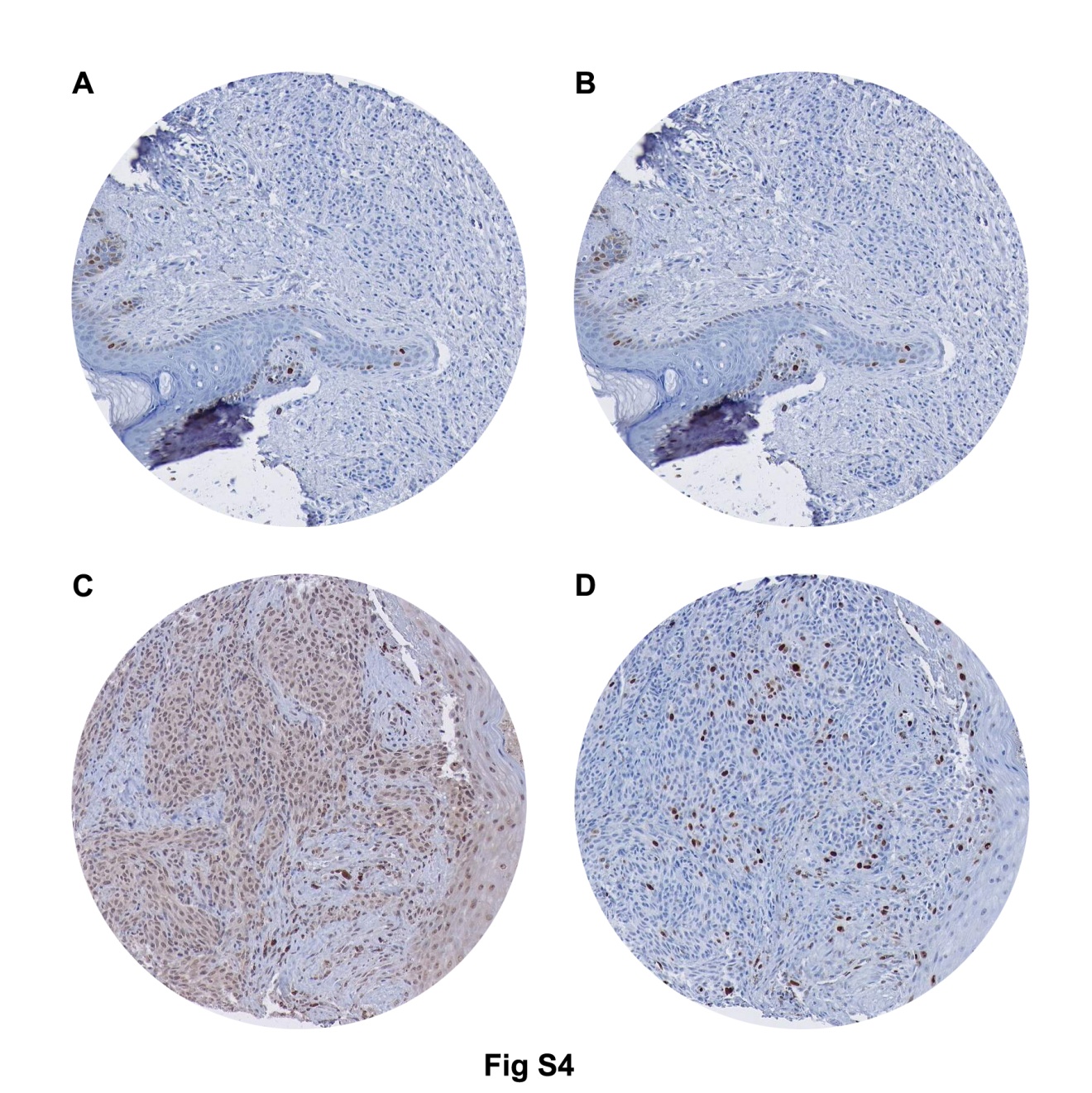

Supplement: Figure S3 — Cytoplasmic Skp2 expression was positively correlated with Ki67 expression. (DOCX) [file pone.0017578.s003.docx]
