# Supplementary material for: Cytoplasmic Skp2 Expression Is Increased in Human Melanoma and Correlated with Patient Survival
Source: PLoS One. 2011 Feb 28;6(2):e17578. doi: 10.1371/journal.pone.0017578 (PMC3046256; doi:10.1371/journal.pone.0017578)
Supplement: Figure S4 — Cytoplasmic Skp2 expression was inversely correlated with nuclear p27 expression. (DOCX) [file pone.0017578.s004.docx]

**Figure S4.** Cytoplasmic Skp2 expression was inversely correlated with nuclear p27 expression. Representative images of cytoplasmic Skp2 (clone A-2, 1:100 dilution; Santa Cruz) (A and C) and nuclear p27 (clone F-8, 1:50 dilution; Santa Cruz) (B and D) staining in human melanocytic lesions. Low cytoplasmic Skp2 staining (A) and high p27 (B) in nevi; High cytoplasmic Skp2 (C) and low nuclear p27 staining (D) in melanoma. Pearson correlation was analyzed between cytoplasmic Skp2 and nuclear p27 staining (n=24) and the coefficient was calculated (r=-0.633, *P*=0.001). Magnification: ×100.

**
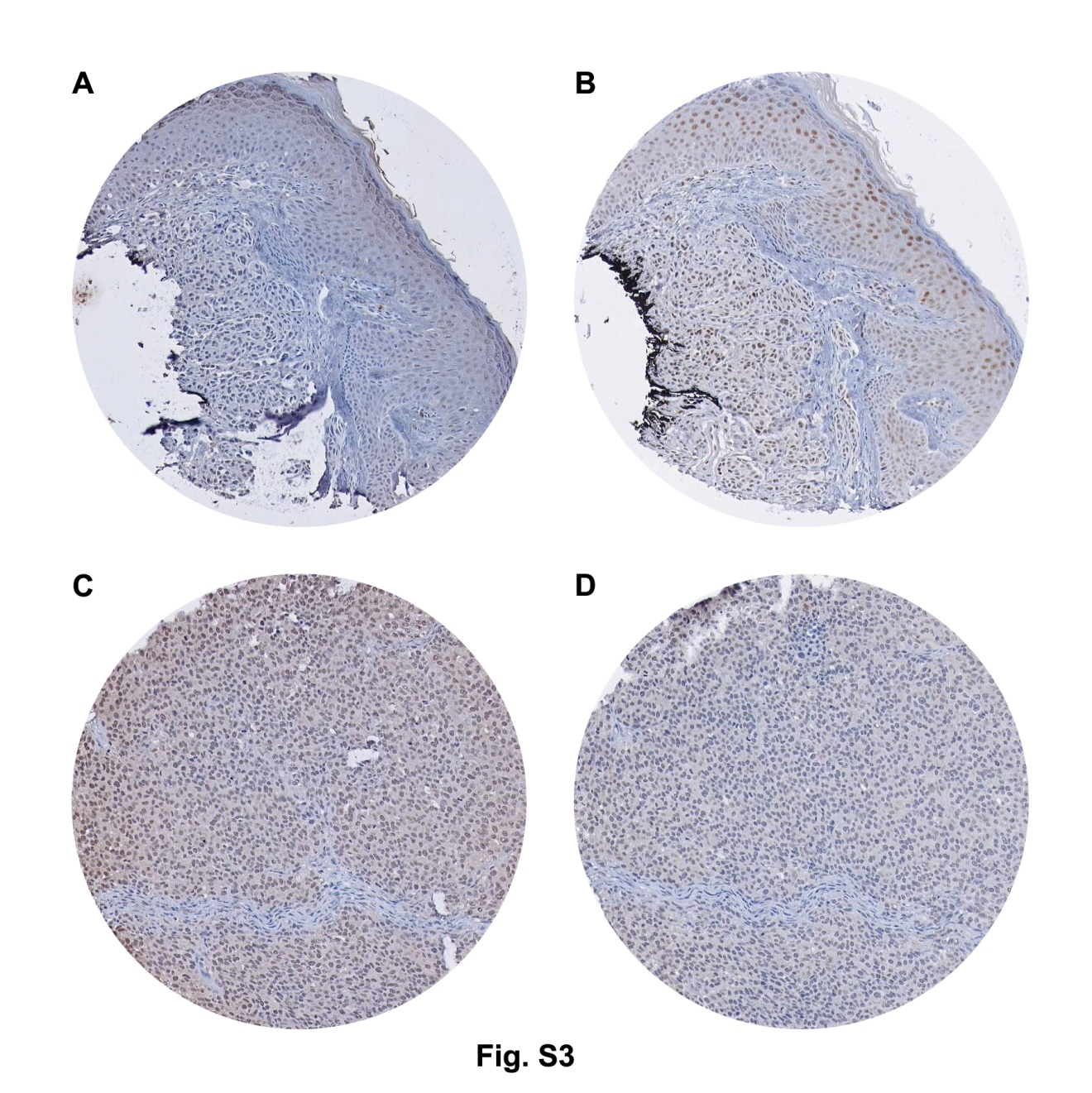
**
