# Supplementary material for: Cytoplasmic Skp2 Expression Is Increased in Human Melanoma and Correlated with Patient Survival
Source: PLoS One. 2011 Feb 28;6(2):e17578. doi: 10.1371/journal.pone.0017578 (PMC3046256; doi:10.1371/journal.pone.0017578)

**Figure S5.** Nuclear Skp2 expression is not associated with melanoma patient survival. Kaplan-Meier curve analyses for the correlation between nuclear Skp2 expression and overall or disease-specific five-year survival in all melanoma patients (A,B), primary melanoma patients (C,D) and metastatic melanoma patients (E,F).


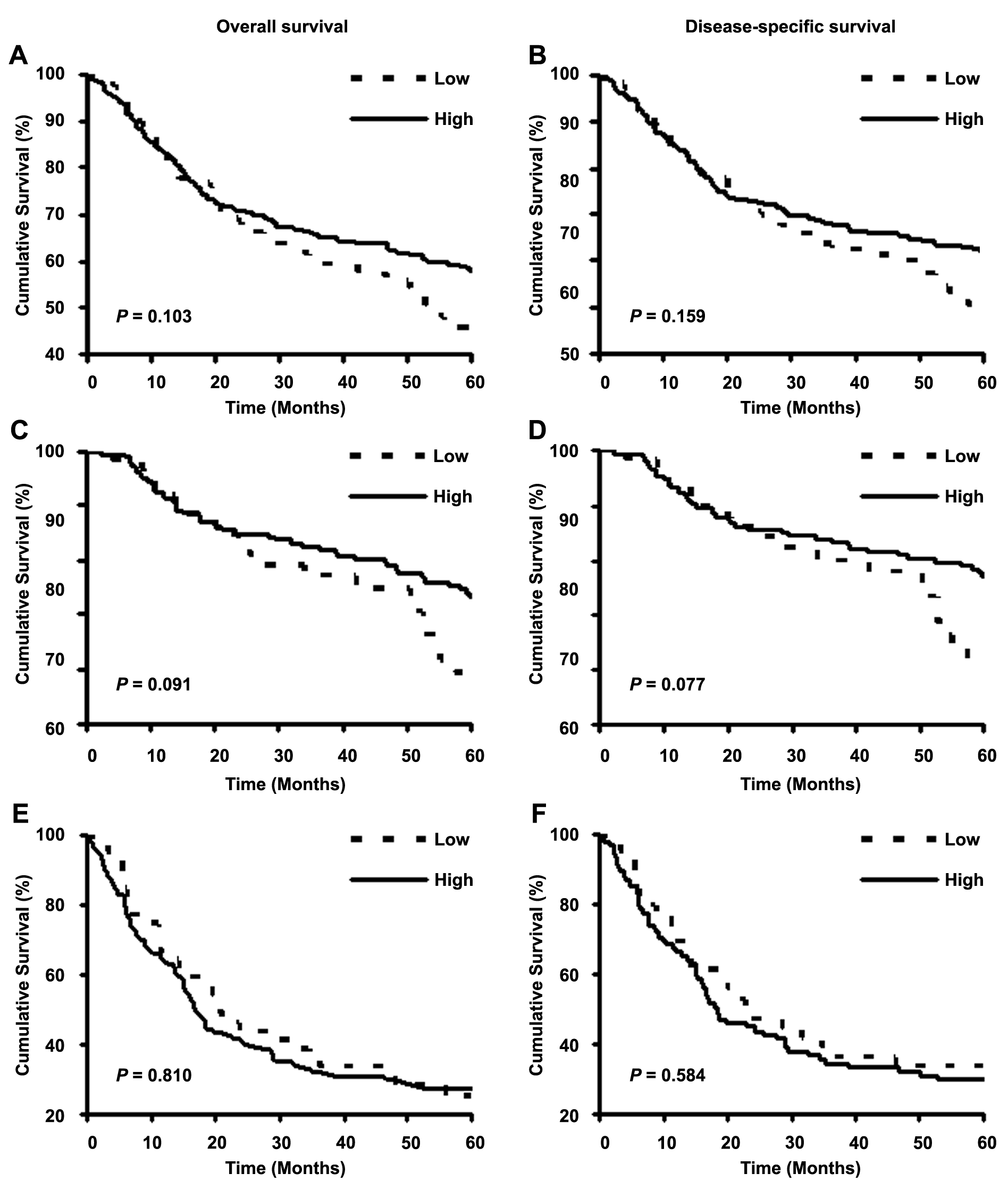

Supplement: Figure S5 — Nuclear Skp2 expression is not associated with melanoma patient survival. (DOCX) [file pone.0017578.s005.docx]
