# Supplementary material for: Cytoplasmic Skp2 Expression Is Increased in Human Melanoma and Correlated with Patient Survival
Source: PLoS One. 2011 Feb 28;6(2):e17578. doi: 10.1371/journal.pone.0017578 (PMC3046256; doi:10.1371/journal.pone.0017578)
Supplement: Table S1 — Univariate Cox proportional regression analysis on overall and disease-specific 5-year survival of all 392 melanoma patients. (DOC) [file pone.0017578.s006.doc]

| **Table S1.** Univariate Cox proportional regression analysis on overall and disease-specific 5-year survival of all 392 melanoma patients | | | | | | | | | | | | | |
| --- | --- | --- | --- | --- | --- | --- | --- | --- | --- | --- | --- | --- | --- |
| **Variable** | **Patients (%)** | **Overall survival** | | | |  | |  | **Disease-specific survival** | | | |  |
|  |  | **Deaths** | **Death Rate** | **HR (95% CI)** | ***P*^†^** | | **Deaths** | | | **Death Rate** | **HR (95% CI)** | ***P*^†^** | |
| Age (years) |  |  |  |  |  | |  | | |  |  |  | |
| ≤ 60 | 201 (51.3%) | 80 | 39.8% | 1.25 (0.92-1.69) | 0.154 | | 78 | | | 38.8% | 1.04 (0.75-1.44) | 0.805 | |
| > 60 | 191 (48.7%) | 86 | 45.0% |  |  | | 70 | | | 36.6% |  |  | |
| Sex |  |  |  |  |  | |  | | |  |  |  | |
| Male | 226 (57.7%) | 101 | 44.7% | 0.87 (0.64-1.19) | 0.434 | | 90 | | | 39.8% | 0.88 (0.63-1.22) | 0.434 | |
| Female | 166 (42.3%) | 65 | 39.2% |  |  | | 58 | | | 34.9% |  |  | |
| AJCC Stage |  |  |  |  |  | |  | | |  |  |  | |
| I | 144 (36.7%) | 21 | 14.6% | 6.13 (3.87-9.71) | <0.001 | | 16 | | | 11.1% | 7.27 (4.32-12.23) | <0.001 | |
| II-IV | 248 (63.3%) | 145 | 58.5% |  |  | | 132 | | | 53.2% |  |  | |
| Cytoplasmic Skp2 |  |  |  |  |  | |  | | |  |  |  | |
| Low expression | 204 (52.0%) | 74 | 36.3% | 1.42 (1.04-1.92) | 0.026 | | 66 | | | 32.3% | 1.41 (1.02-1.96) | 0.036 | |
| High expression | 188 (48.0%) | 92 | 48.9% |  |  | | 82 | | | 43.6% |  |  | |
| Nuclear Skp2 |  |  |  |  |  | |  | | |  |  |  | |
| Low expression | 166 (42.3%) | 50 | 30.1% | 0.76 (0.55-1.06) | 0.104 | | 44 | | | 26.5% | 0.78 (0.55-1.11) | 0.160 | |
| High expression | 226 (57.7%) | 116 | 51.3% |  |  | | 108 | | | 47.8% |  |  | |
| ^†^Log-Rank test  Abbreviations: HR, hazard ratio; CI, confidence interval. | | | | | | | | | | | | | |
