# Supplementary material for: Cytoplasmic Skp2 Expression Is Increased in Human Melanoma and Correlated with Patient Survival
Source: PLoS One. 2011 Feb 28;6(2):e17578. doi: 10.1371/journal.pone.0017578 (PMC3046256; doi:10.1371/journal.pone.0017578)
Supplement: Table S2 — Univariate Cox proportional regression analysis on overall and disease-specific 5-year survival of 133 patients with metastic melanoma. (DOC) [file pone.0017578.s007.doc]

| **Table S2.** Univariate Cox proportional regression analysis on overall and disease-specific 5-year survival of 133 patients with metastatic melanoma | | | | | | | | | | | | | |
| --- | --- | --- | --- | --- | --- | --- | --- | --- | --- | --- | --- | --- | --- |
| **Variable** | **Patients (%)** | **Overall survival** | | | |  | |  | **Disease-specific survival** | | | |  |
|  |  | **Deaths** | **Death Rate** | **HR (95% CI)** | ***P*^†^** | | **Deaths** | | | **Death Rate** | **HR (95% CI)** | ***P*^†^** | |
| Age (years) |  |  |  |  |  | |  | | |  |  |  | |
| ≤ 60 | 77 (57.9%) | 59 | 76.7% | 0.90 (0.60-1.36) | 0.627 | | 57 | | | 74.0% | 0.77 (0.50-1.19) | 0.239 | |
| > 60 | 56 (42.1%) | 38 | 67.9% |  |  | | 31 | | | 55.4% |  |  | |
| Sex |  |  |  |  |  | |  | | |  |  |  | |
| Male | 88 (66.2%) | 64 | 72.7% | 1.12 (0.74-1.71) | 0.599 | | 56 | | | 63.6% | 1.24 (0.80-1.91) | 0.334 | |
| Female | 45 (33.8%) | 33 | 73.3% |  |  | | 32 | | | 71.1% |  |  | |
| Cytoplasmic Skp2 |  |  |  |  |  | |  | | |  |  |  | |
| Low expression | 59 (44.4%) | 44 | 74.6% | 0.93 (0.62-1.39) | 0.714 | | 41 | | | 69.5% | 0.89 (0.58-1.35) | 0.572 | |
| High expression | 74 (55.6%) | 53 | 71.6% |  |  | | 47 | | | 63.5% |  |  | |
| Nuclear Skp2 |  |  |  |  |  | |  | | |  |  |  | |
| Low expression | 39 (29.3%) | 29 | 74.4% | 1.06 (0.68-1.63) | 0.810 | | 25 | | | 64.1% | 1.14 (0.72-1.81) | 0.585 | |
| High expression | 94 (70.7%) | 68 | 72.3% |  |  | | 63 | | | 67.0% |  |  | |
| ^†^Log-Rank test  Abbreviations: HR, hazard ratio; CI, confidence interval. | | | | | | | | | | | | | |
