# Supplementary material for: Cytoplasmic Skp2 Expression Is Increased in Human Melanoma and Correlated with Patient Survival
Source: PLoS One. 2011 Feb 28;6(2):e17578. doi: 10.1371/journal.pone.0017578 (PMC3046256; doi:10.1371/journal.pone.0017578)
Supplement: Table S3 — Multivariate Cox regression analysis on overall and disease-specific 5-year survival of all 392 melanoma patients. (DOC) [file pone.0017578.s008.doc]

| **Table S3.** Multivariate Cox regression analysis on overall and disease-specific 5-year survival of all 392 melanoma patients | | | | | | | | | | | |
| --- | --- | --- | --- | --- | --- | --- | --- | --- | --- | --- | --- |
| **Variables*** | **Overall survival** | | | | |  | **Disease-specific survival** | | | | |
|  | **    β^†^** | **SE** | **HR** | **95% CI** | ***P*** |  | **      β^†^** | **SE** | **HR** | **95% CI** | ***P*** |
| Age | 0.041 | 0.157 | 1.04 | 0.77-1.42 | 0.792 |  | -0.148 | 0.166 | 0.86 | 0.62-1.19 | 0.373 |
| Sex | 0.097 | 0.161 | 1.10 | 0.80-1.51 | 0.547 |  | 0.116 | 0.170 | 1.12 | 0.80-1.57 | 0.496 |
| AJCC | 1.831 | 0.241 | 6.24 | 3.89-10.02 | <0.001 |  | 2.031 | 0.273 | 7.62 | 4.47-13.01 | <0.001 |
| Cytoplasmic Skp2 expression | -0.028 | 0.160 | 0.97 | 0.71-1.33 | 0.864 |  | -0.031 | 0.170 | 0.97 | 0.70-1.35 | 0.857 |
| *Coding of variables: Age was coded as 1 (≤60 years), and 2 (>60 years). Sex was coded as 1 (male) and 2 (female). AJCC was coded as 1 (Stages I-II) and 2 (Stages III-IV). Cytoplasmic Skp2 expression was coded as 1 (low staining) and 2 (high staining).  ^†^β: regression coefficient.  Abbreviations: SE, standard error of β; HR, hazard ratio; CI, confidence interval. | | | | | | | | | | | |
